# Feeling Weary? Feeling Insecure? Are All Workplace Changes Bad News?

**DOI:** 10.3390/ijerph16101842

**Published:** 2019-05-23

**Authors:** Irina Nikolova, Karen van Dam, Joris Van Ruysseveldt, Hans De Witte

**Affiliations:** 1 Faculty of Management, Science and Technology, Open University, Valkenburgerweg 177, 6419 AT Heerlen, The Netherlands; 2 Research group for Work, Organisational and Personnel Psychology, KU Leuven, Dekenstraat 2, 3000 Leuven, Belgium; hans.dewitte@kuleuven.be; 3 Faculty of Psychology & Educational Sciences, Open University, Valkenburgerweg 177, 6419 AT Heerlen, The Netherlands; karen.vandam@ou.nl (K.v.D.); joris.vanruysseveldt@ou.nl (J.V.R.); 4 Optentia Research Focus Area, Vanderbijlpark Campus, North-West University, Hendrik Van Eck Blvd, Vanderbijlpark 1900, South Africa

**Keywords:** workplace changes, learning demands, qualitative job insecurity, competence development, emotional exhaustion

## Abstract

Prior research indicates that workplace changes can have both positive and negative consequences for employees. To explore the mechanisms that trigger these different outcomes, we propose and test a mediation model, which builds on the premises of the challenge–hindrance model of work stress. Specifically, we suggest that whereas workplace changes can engender positive outcomes (e.g., learning outcomes) through an increase in learning demands, they can also enhance negative outcomes (e.g., emotional exhaustion) through increased perceptions of qualitative job insecurity. While we made these specific assumptions, we also analyzed the reversed causation relationships. Two-wave data obtained from 1366 Dutch employees were used to test the study hypotheses. The results showed that the reciprocal causation model had the best fit for the data. However, whereas emotional exhaustion was only mediated by qualitative job insecurity, no mediation was found by learning demands. In addition to the hypothesized effects, several reversed causation effects emerged from the analyses, indicating that the relationships between workplace changes and employee learning and strain are not unidirectional. This underscores the need for a broader view on the causes and effects of workplace changes, as the traditional causation relationships (i.e., perceptions of workplace changes impacting employee learning and strain experiences) are insufficient to explain the complex dynamics between the studied phenomena.

## 1. Introduction

Workplace changes have become a ubiquitous part of today’s working life. As organizations need to continuously adapt to dynamic and competitive business environments, this impacts employees’ functioning at work including their competence development and well-being [1,2,3]. 

The objective of this study is to provide an explanation for both favourable and detrimental outcomes of workplace change, while at the same time investigating theoretical paths that can explain these different outcomes. We used the challenge–hindrance model of work stress [4,5] as our primary theoretical framework. We propose two processes that are likely to shape the relationships between workplace changes and employee outcomes: a motivational process by which workplace changes increase the demand for work-related learning (operating as a challenge stressor) and subsequently stimulate employees to obtain new knowledge and skills [5], and an energy-depletion process by which workplace changes enhance qualitative job insecurity (operating as a hindrance stressor) and subsequently increase employee exhaustion. In sum, we argue that workplace changes can trigger both negative outcomes (e.g., emotional exhaustion) and positive outcomes (e.g., learning outcomes, meaning the acquisition of new work-related competencies as a result of engagement in learning processes), because these changes initiate or reinforce specific challenging and hindering work aspects. Research has highlighted two work characteristics to be highly responsive to workplace changes: qualitative job insecurity (i.e., anticipated loss of the *valued aspects* of one’s job; [6]), which is a hindrance stressor [4,6], and learning demands, which are a challenge stressor [7,8]. 

Whereas prior studies on the consequences of organizational change on employees have mainly focused on changes such as downsizing, acquisitions, and organizational restructuring, much less research has focused on workplace changes (i.e., shifts in (1) production methods and ways of working; (2) technology such as automatization and digitalization; and (3) production procedures, standards, and regulations [9], and the way they affect employees [10]). This underscores the importance of gaining insights into the *mechanisms* through which workplace changes might relate to positive and negative outcomes for employees (e.g., learning and exhaustion), as empirical research thus far has provided little evidence on *how*, in fact, employee learning and strain at work come about. Considering that workplace changes impact the very nature of the work and the context in which a job is performed, it is important to generate more knowledge on the processes that evolve from workplace changes and how these changes influence employee functioning at work. 

In academic literature, the consequences of workplace changes have been studied in different fields linking change to a variety of employee and organizational outcomes. In the field of organizational change, change is generally considered a work stressor [11] that is associated with increased employee strain [12,13,14,15]; work strain experiences can result from a change-induced rise in job demands such as job insecurity [1] and the interruption of employees’ daily work routines [16]. In the field of workplace learning, however, researchers have emphasized that workplace changes require employees to develop new knowledge, skills, and work routines and can therefore increase employee learning [17,18,19]. Moreover, several scholars [17,18,20] have suggested that exposure to changes can be particularly beneficial for workplace learning, because jobs affected by frequent changes in technology and working methods are more “learning-intensive” by nature. Altogether, research within the organizational change domain has provided initial evidence on both the strain [2,15] and the learning [18] perspective; however, as far as we know, organizational change researchers have not yet tried to integrate these two perspectives, nor have researchers attempted to explain the mechanisms through which workplace learning and strain might evolve as outcomes of change implementation. 

Using the challenge–hindrance model of work stress [4,5], we propose that hindrance stressors (e.g., increased qualitative job insecurity) and challenge stressors (e.g., increased learning demands) result in increased emotional exhaustion. Additionally, we assume that qualitative job insecurity—as a hindrance work aspect—is a cause of frustration, while learning demands—as a challenge work aspect—advances learning outcomes. In the current contribution, we focused on qualitative job insecurity as a mediator in the relationship between workplace changes and the two study outcomes. We chose to explore the role of qualitative but not quantitative job insecurity (i.e., fear of losing a current job in the near future [6]) for two reasons. 

First, from a theoretical point of view, it seems particularly relevant to study how workplace changes that directly affect the nature of a person’s daily work (because they are aimed at alternations in work methods, processes, and technology) can impact employees’ perceptions of anticipated loss of *valued job aspects.* Such impact is likely because employees might experience reduced control over the change process (inherent to the top-down nature of the change implementation), including the inability to control and retain valuable job aspects. As quantitative job insecurity refers to the fear of job loss, it seems less plausible that workplace changes (i.e., changes in work methods, processes, and technology) would trigger anxieties regarding the (dis)continuation of employment. 

Second, from a methodological point of view, the choice to examine the role of qualitative job insecurity (and not quantitative) is justified because our study sample consisted predominantly of employees who hold a permanent contract. Whereas temporary workers might be less concerned with the possible loss of valuable aspects of their job in the future (as they are less likely to build high expectations regarding the future of their current employment), permanently employed individuals are likely to be particularly vulnerable to the effects of qualitative job insecurity. The permanent nature of their employment implies that potential changes in valuable aspects of their jobs can have a long-lasting impact on their functioning at work. Moreover, temporary workers might be more preoccupied with fears of losing their job (and less with worries about the future quality of their job). Several studies have indicated that shared concerns regarding quantitative job insecurity are more likely to emerge in organizations with a high representation of temporary workers [21,22]. 

Our study aims to contribute to the literature in three major ways. First, by building on the theoretical assumptions of the challenge–hindrance framework [4,5], we attempted to explain the different processes that evolve from workplace changes and to provide a rationale for the positive and negative consequences of these changes. By selecting one challenge stressor (learning demands) and one hindrance stressor (qualitative job insecurity), which in previous research were shown to be associated with or initiated by organizational change [20], we shed light on two processes that unfold from workplace changes: a learning and a strain process. 

Second, we linked workplace changes to qualitative job insecurity because we wanted to clarify how top-down changes (regarding work methods, processes, and technology) relate to employees’ anticipation of losing valuable job aspects. This is key as employees’ perceived limited control over the change process (inherent to the top-down nature of the change implementation) might well translate into a perceived inability to control and retain valuable job aspects such as favourable working conditions, wage, career opportunities, and interesting work content. 

Third, our study aims to contribute to the workplace learning [10,20] and thriving at work [23] literature. It does so by expanding our insight into the role of organizational change in workplace learning and its underlying processes. We expect workplace change to reinforce challenging work aspects (e.g., learning demands), which result in increased learning outcomes. However, by including qualitative job insecurity as a hindrance stressor in our model, we also investigated an alternative pathway with a potentially negative impact on learning. The effect of hindrance stressors on workplace learning has received little attention in research [23]. 

### 1.1. Learning Demands and Qualitative Job Insecurity in Relation to Workplace Changes

Workplace changes, especially when they concern ample shifts in several core aspects of an organization, such as the working methods, production processes, and technology [11,24], could have a high impact on the nature of work and the way the employees perform their job. As such, workplace changes can result from both episodic and continuous change [25]. Episodic change is infrequent, discontinuous and intentional and is decided at the strategic level of the organization; continuous change is generally recurrent, cumulative, and emergent and is initiated at a lower level. Whether episodic or continuous, workplace changes as a common work stressor could imply a considerable shift in some of the core aspects of an organization [11,24] and consequently could have a large impact on employees’ behaviour, attitudes, and well-being [1,3]. Such changes may thus affect a number of key job aspects (both challenging and hindering by nature) in the workplace. We propose that owing to changes in how daily work tasks are performed, workplace change can engender increased demands for learning at work—a challenging work aspect—and can increase employee perceptions of qualitative job insecurity—a hindering work aspect. 

Altogether, workplace changes are considered an important impetus for workplace learning [10,17] owing to increased learning demands. Learning demands refer to the employees’ experience of pressure to obtain new work-related competences [8]. Changing working conditions resulting from shifts in work routines, methods, and technology may require employees to gain the knowledge and skills that are necessary to perform their jobs effectively [8,19]. The fast pace of environmental and workplace changes generally means employees need to continuously adapt their skills and knowledge in order to keep up their performance and secure their position on the labour market [26,27]. Changes in working methods, procedures, or tools may imply that the knowledge and skills used previously for carrying out daily tasks may become obsolete and insufficient [18] and that employees need to adapt their knowledge, skills, and work routines to retain their level of competence [17]. In other words, workplace changes may result in increased learning demands that foster learning and professional development [28]. In fact, changes such as job transitions and challenging assignments are deliberately used in employee development programs to stimulate the learning demands of employees and managers [29]. Learning demands can be considered a challenge because they contribute to personal development and growth and thus constitute a motivational force; yet, meeting these demands may require substantial effort and stretch an individual’s abilities [23]. In sum, employees in a situation of workplace changes are likely to face an increase in learning demands owing to a possible mismatch between current and required competencies [19].

**Hypothesis** **1:** *Workplace changes are positively related to learning demands.*

In addition, workplace changes may trigger perceptions of qualitative job insecurity [30]. That is, changes in working methods, technology, and production processes may cause employees to develop concerns regarding the continued existence of valued aspects of their jobs, such as job characteristics (e.g., autonomy, responsibility), colleagues, status, and career progress [31,32]. Insecurities about valued job aspects are considered to be innate to organizational change in general and workplace changes in particular, as the process and outcome of change implementation are often unclear [1,33]. According to Ashford and colleagues [34], change is one of the main antecedents of perceived job insecurity because it undermines perceptions of control and increases role ambiguity and role conflict. Since some job aspects can be particularly valuable for the individual, a perceived threat to these aspects such as the threat posed by workplace changes (a general work stressor) may enhance experiences of qualitative job insecurity among employees and may, in time, tax their well-being [35,36]. Job insecurity has been repeatedly identified as a hindering work aspect that increases employee strain and negative emotions and reduces work goal achievement [30,37]. In sum, as workplace changes are implemented from the top down [38] and are aimed at changing the way work is carried out, they are likely to be perceived as beyond employees’ control and thus may enhance their *fears* of losing valued job aspects (i.e., qualitative job insecurity [33]).

**Hypothesis** **2:** *Workplace changes are positively related to qualitative job insecurity.*

### 1.2. Outcomes of Learning Demands and Qualitative Job Insecurity

We propose that learning demands and qualitative job insecurity have some similarities as well as differences in the way they impact the study outcomes. We expect learning demands to increase emotional exhaustion and learning outcomes, and qualitative job insecurity to result in higher emotional exhaustion but fewer learning outcomes. 

Theoretically, these assumptions about the relationships between learning demands and qualitative job insecurity on the one side and the two study outcomes on the other are derived from the challenge–hindrance framework of work stress. This framework distinguishes between two types of work stressors according to their potential to either support (challenge stressors) or obstruct (hindrance stressors) employees’ goal attainment [4,35]. Hindrance stressors, such as qualitative job insecurity, may elicit a typical stress response, with high arousal and negative emotions, that can result in avoidance behaviours and health impairment such as emotional exhaustion and physical complaints [5,39]. Challenging stressors, such as learning demands [23], may similarly require high levels of arousal and information processing and can therefore result in energy depletion [40]. At the same time, as they hold the potential to promote personal gain or growth, challenging stressors may trigger positive emotions and cognitions and increased motivation and approach behaviours [41]. The distinction between the consequences of challenge versus hindrance stressors has received considerable empirical support in previous research [41,42,43,44]. 

As learning demands and qualitative job insecurity both represent demanding work conditions (or job demands), substantial effort and information processing (i.e., state of activation) are needed for the individual to deal with them; sustained activation can drain a person’s energy and result in emotional exhaustion [43]. Emotional exhaustion is an important component of burnout and pertains to work-related experiences of emotional fatigue and weariness caused by the depletion of the individual’s resources [45]. Prior research convincingly showed that, over time, sustained activation evoked by challenge and hindrance stressors is likely to result in experiences of considerable energy loss and fatigue, which in turn can result in emotional exhaustion [5,46].

**Hypothesis** **3:** *Learning demands are positively related to emotional exhaustion.*

**Hypothesis** **4:** *Qualitative job insecurity is positively related to emotional exhaustion.*

We expect learning demands and qualitative job insecurity, as challenge and hindrance stressors, to differ in their effects on learning outcomes [5]. While learning demands boost employee learning outcomes, qualitative job insecurity is likely to decrease learning. Empirical evidence on these relationships is still scarce. To date, only a handful of studies have explored the link between learning demands and employee outcomes [47,48]. Moreover, to the best of our knowledge, only few studies have investigated how learning demands positively affected employee learning [47,48]. In their study, Prem and colleagues [23] found that learning demands directly increased learning at work. Paulsson et al. [49] concluded that knowledge requirements at work (i.e., the requirement for workplace skills and competences that demand employee learning, a concept close to learning demands) stimulated competence development and learning outcomes. In sum, we assume that:

**Hypothesis** **5:** *Learning demands are positively related to learning outcomes.*

Similarly, few studies have covered the impact of qualitative job insecurity on learning outcomes. The link between quantitative job insecurity and the willingness to undertake training has recently been studied [50]. Also, Elman and O’Rand [51] have previously studied the association between perceived job insecurity and actual training participation. However, to the best of our knowledge, no study has been published on the association of qualitative job insecurity with learning outcomes, such as acquiring new skills and competencies. We assume a negative association between qualitative job insecurity and learning outcomes, because worries about a future job situation may take away the attention and direction an employee needs for learning activities [52]. Worries, anxieties, and stress arousal may place a high demand on employees’ cognitive resources and thus interfere with effective learning [52,53,54]. Research on challenge and hindrance stressors has shown that hindrance stressors can negatively impact learning motivation and learning performance [5]. We thus hypothesize that:

**Hypothesis** **6:** *Qualitative job insecurity is negatively related to learning outcomes.*

In summarizing, we propose that workplace changes will result in increased learning demands and increased qualitative job insecurity. In line with the challenge–hindrance stressors framework, we expect that learning demands will lead to both increased emotional exhaustion and increased learning outcomes. Similarly, we propose that qualitative job insecurity will result in increased emotional exhaustion. Finally, and in addition to the challenge–hindrance stressors framework, we assume that qualitative job insecurity will lead to decreased learning outcomes. 

Together, these hypotheses suggest that workplace changes initiate two processes, a learning and a strain process. While previous research has already shown that workplace changes can lead to learning [29] and strain [11], this study emphasizes the role of learning demands and qualitative job insecurity in these relationships. In addition to the indirect relationships that are expressed in the hypotheses, we pose that learning demands and qualitative job insecurity serve as mediators in the learning and strain processes. Within the stress and change literature, different models have been proposed that describe how events, such as change, exert an effect through perceptions, emotions, and attitudes (e.g., Lazarus & Folkman, 1994 [55]; Oreg et al., 2018 [56]; Rafferty & Griffin, 2006 [11]). Lazarus and Folkman’s appraisal theory [55], for instance, delineates how an experience initiates a process of appraisals that subsequently gives rise to emotional responses, which in turn can have a behavioural or health effect. In line with the appraisal theory, we argue that work place changes cause perceptions of increased learning demands and subsequently result in (more or less) learning outcomes and emotional exhaustion.

**Hypothesis** **7:** *Learning demands mediate the relationships of workplace changes with learning outcomes (H7a) and emotional exhaustion (H7b).*

**Hypothesis** **8:** *Qualitative job insecurity mediates the relationships of workplace changes with learning outcomes (H8a) and emotional exhaustion (H8b).*

### 1.3. Temporality

In this study, we explored the link between workplace changes and their associates (i.e., learning demands and qualitative job insecurity) at the same time point (T1) and we tested their effects on the study outcomes (i.e., emotional exhaustion and learning outcomes) over a period of six months (T2). We opted to test workplace changes and the related hindrance and challenge appraisals of the changing situation synchronously. We thus looked at whether the changes require employee learning and increase feelings of insecurity. Stress theories (e.g., Lazarus & Folkman, [55]) suggest that evaluative mechanisms and responses are triggered nearly instantly when the individual is confronted with a stressor (in our case, workplace changes). Synchronous effects between the independent variable and the study mediators are likely because these variables operate in the same cognitive and temporal space [57,58]. That is, work change as a stressor may have an immediate impact on perceptions of learning demands and qualitative job insecurity. As Wong and Law [58] noted, synchronous effects do not imply that these effects are simultaneous, rather that the exact time lags between constructs are either too short to capture, unknown, or impractical in terms of measurement. In line with previous studies investigating the effects of work stress on employees’ strain and learning [59,60,61], we chose a time lag of approximately six months to assess exhaustion and learning as outcomes evolving from work restructuring and its associates (i.e., learning demands and qualitative job insecurity). In addition, Job Demands-Resources (JD-R) research also provided theoretical rationale and empirical support for the suitability of longer time lags (e.g., of a few months) when testing the effect of job characteristics on employees’ psychological states [62]. The idea that longer time lags are needed to measure the effects of the work context on a person’s psychological state rests on the assumption that “job characteristics and social relationships tend to be somewhat inert and typically cannot be changed at short notice (Hackman & Oldham, 1980; Parker et al., 2003)” [62].

## 2. Materials and Methods

### 2.1. Procedure and Sample

Data were collected by an ISO-certified online marketing research company (the company is registered under the Dutch Data Protection Authority (CBP) in The Hague) that had access to a large group of Dutch employees working in multiple organizations who have agreed to take part in online survey data collections. Each time they participated in the survey, the employees earned 100 points (which equalled a modest financial reward). 

At the end of March 2012 (T1), approximately 3500 wage earners in the age category between 18 and 64 years of age received an online invitation to fill out the questionnaire. Three days after the initial invitation, an e-mail was sent out to remind the participants to complete the survey; the questionnaire was made available online to the employees for a one-week period. To obtain a sample that was close to the representative sample for the Dutch working population, several filters were used when selecting the respondents. Demographic preselection (i.e., gender, age, education, and geographic location) was conducted in line with the “Golden Standard” (a calibration tool), which was developed to keep marketing companies informed about the most recent national statistics for the Netherlands, provided by the Central Agency for Statistics (CBS; [63]). The respondents completed the survey voluntarily and could discontinue their participation at any point during data collection; they were informed that the data would be used for research purposes only, that the collected data would be handled confidentially, and no identifying personal information (e.g., names or contact information) would be made available to the researchers or other parties. The respondents who completed the questionnaire at T1 (*n* = 1711, 49% response rate) were invited to take part in the second data collection, which took place six months later (T2). At the beginning of October 2012, a total of 1366 respondents completed the questionnaire at T2 (81% response rate). Similarly to the procedure followed during the first data collection, a reminder was sent three days after the initial invitation. Each participant was assigned a unique survey number (code), which was used during the two points of data collection. Later, each participant’s responses were matched over time using this code. This sample was used to test the hypotheses. Mean age was 44.25 years (*SD* = 10.89); 59.2% were male. Most respondents had a permanent contract (89.6%); educational level ranged from lower educational training (16.5%), to mid-level educational training (45.2%) and higher educational training (38.3%).

Attrition analyses with crosstabs indicated that the respondents at T2 were more often male (59.2% vs. 57.9%, *p* < 0.05) and held a higher educational degree (38.3% vs. 37.1%, *p* < 0.05). Logistic regression showed that drop-outs were a little younger (*OR* = 1.02, *p* < 0.01; (*χ*^2^(1) = 9.23, *p* < 0.01) and experienced somewhat more qualitative job insecurity (*OR* = 0.83, *p* < 0.05; *χ*^2^(6) = 9.23, *p* < 0.001). 

### 2.2. Measures

Workplace changes. The extent of workplace changes was assessed with three items adapted from scales on work changes and innovation developed by Jiménez-Jiménez and Sanz-Valle [64]. Items were preceded by the following text “In my department, during the past six months, changes occurred regarding …”. A sample item was “… the work practices for producing goods or delivering services”. A five-point response scale was used, ranging from 1 (*to a very small degree*) to 5 (*to a very large degree*). Cronbach’s alpha was 0.80 at T1 and 0.81 at T2. 

Learning demands. Learning demands were measured with three items adapted from research by Mikkelsen, Øgaard, and Landsbergis [65] and Shih et al. [48]. An example item was: “My job compels me to learn new things.” The responses could be indicated on a five-point Likert scale ranging from 1 (*to a very small degree*) to 5 (*to a very large degree*). Cronbach’s alpha was 0.91 at T1 and 0.92 at T2. 

Qualitative job insecurity. To measure qualitative job insecurity, a three-item scale was used, tapping into similar aspects as the items of De Witte and colleagues [66]. This scale was previously used in Roll, Siu. and Li [67] and Van den Broeck et al. [68]. A sample item was “I feel uncertain about the content of my job in the future”. A five-point response scale was used, ranging from 1 (*strongly disagree*) to 5 (*strongly agree*). Cronbach’s alpha was 0.88 at T1 and 0.89 at T2. 

Learning outcomes. Learning outcomes were measured with a four-item scale developed by Taverniers [69] that has shown good reliability and validity in previous studies [9]. A sample item was “In the past six months, I have obtained new competences, which help me to conduct my work more efficiently”. The response scale ranged from 1 (*strongly disagree*) to 5 (*strongly agree*). Cronbach’s alpha was 0.95 for both T1 and T2. 

Emotional exhaustion. Emotional exhaustion was assessed with the five-item emotional exhaustion scale of the Maslach Burnout Inventory (MBI) [45]). A sample item was “I feel “burned out” by my work”. The response scale ranged from 1 (*never*) to 7 (*always*). Cronbach’s alpha was 0.93 for both T1 and T2. 

### 2.3. Analyses

Testing the measurement model. Prior to testing the study hypotheses, we carried out preliminary analyses to establish the robustness of the suggested five-factor model. In addition to Confirmatory Factor Analyses (CFA), the measurement invariance of the model across time was tested. Several indices were used to determine the goodness of fit of the models [70]: chi-square (χ^2^), root-mean-square errors of approximation (RMSEA ≤ 0.08), standardized root-mean-square residual (SRMR ≤ 0.08; Hu & Bentler, 1999), comparative fit index (CFI ≥ 0.90), the Tucker–Lewis index (TLI ≥ 0.90), the Akaike Information Criterion (AIC), and the Bayesian Information Criterion (BIC). AIC and BIC indicate the balance between the number of parameters—that is, the model complexity—and the fit of the model to the data, where lower values indicate a better fit. Furthermore, CFI difference was used for model comparison (Cheung and Rensvold, 2002) [71]. 

Testing the research model. Latent factor structural equation modelling (in Mplus 7.4; [72]) was used to test the study hypotheses. Each of the study variables was specified as a latent factor measured at two time points (T1 and T2). For a part of the analyses, we followed the technique recommended by Cole and Maxwell [73] to analyse cross-lagged relationships between the predictor and the outcomes and between the mediators and the outcomes. Subsequently, we conducted a full panel of mediation analyses to test indirect effects, multiplying the a-paths of our model with the b-paths. We obtained indirect effects for the *reciprocal full-panel model*, as the results from the analyses (described above) suggested that the reciprocal model might be the most suitable model to explain the relationships between the study variables.

According to the recommendations of Cole and Maxwell [73] (see also [74,75]), several sets of cross-legged analyses (i.e., stability, causal, reversed, and reciprocal models) need to be performed to establish semi-longitudinal mediation. In line with this method, we examined the cross-lagged relationships between the predictor “workplace changes” and the two outcome variables “learning outcomes” and “emotional exhaustion” and between the two mediators “learning demands” and “qualitative job insecurity” and the study outcomes “learning outcomes” and “emotional exhaustion”. Since no temporal effects between the study predictor and mediators were assumed (i.e., workplace changes were expected to influence employee perceptions of learning demands and qualitative job insecurity much sooner than within six months), no cross-lagged relationships between these variables were explored. Instead, we tested a normal and a reversed causation between the predictors at T2 and the mediators at T2 to explore the significance of these relationships.

To test the cross-lagged relationships between the predictor and the outcomes and between the mediators and the outcomes, several competing models were specified: a *stability model* (M_stabil_) where autoregressive paths between each pair of latent constructs across time were modelled; a *causality model* (M_causal_) where the causal relationships (for M1_causal_ between the predictor and the two study outcomes, and for M2_causal_ between the two mediators and the two study outcomes) were added to the stability model; a *reversed causation model* (M_revers_) where the reversed hypothesized relationships (for M1_revers_ between the two outcomes and the predictor, and for M2_revers_ between the two outcomes and two mediators) were added to the stability model; a *reciprocal model* (M_recipr_) in which all paths specified in M_stabil_**,** M_causal_, and M_revers_ were included (i.e., M1_recipr_ comprised M1_stabil_, M1_causal_, and M1_revers_, and M2_recipr_ comprised M2_stabil_, M2_causal_, and M2_revers_). A *χ*^2^ -difference test was used to compare the proposed competing models (i.e., stability, causal, reversed, and reciprocal), in addition to the earlier described goodness-of-fit indices (RMSEA, TLI, and CFI; see Byrne [70]).

## 3. Results

Table 1 presents the correlations, means, and standard deviations of the study variables.

### 3.1. Testing the Measurement Model

To test the robustness of our measurement model, we first analyzed a model including all study constructs at T1 and T2 simultaneously. This model showed an acceptable fit for the data (χ^2^ (*df* = 620) = 2424.48; *p* < 0.001; RMSEA = 0.05; CFI = 0.96; TLI = 0.95; BIC = 119463.52). Factor loadings ranged from 0.66 to 0.83 (T1) and from 0.69 to 0.83 (T2) for workplace changes; from 0.86 to 0.89 (T1) and from 0.87 to 0.91 (T2) for learning demands; from 0.76 to 0.83 (T1) and from 0.79 to 0.84 (T2) for qualitative job insecurity; from 0.87 to 0.94 (T1) and from 0.88 to 0.94 (T2) for learning outcomes; and from 0.81 to 0.87 (T1) and from 0.80 to 0.90 (T2) for emotional exhaustion. Together, these results indicated that the scales in our study measured distinct constructs and could be used to analyze the research model.

In addition, we tested whether the study five-factor model was invariant across the two waves by restricting the measurement model and comparing the model fit in several steps. In line with the recommendations of Cheung and Rensvold [76], a decrease in the CFI greater than 0.01 indicated a meaningful decrement in fit. First, we evaluated an unconstrained stability model (χ^2^(620) = 2424.48, *p <* 0.001, CFI = 0.96, TLI = 0.95, AIC = 118, 633.59, BIC = 119,463.52, RMSEA = 0.05, SRMR = 0.03). Next, we constrained the factor loadings of the respective factors to be equal across T1 and T2 (χ^2^(634) = 2442.36, *p <* 0.001, CFI = 0.96, TLI = 0.95, AIC = 118, 623.48, BIC = 119,380.33, RMSEA = 0.05, SRMR = 0.03). Compared to the unconstrained model, the model with constrained factor loadings did *not* show a significant decrease of CFI (ΔCFI < 0.01). This non-significant loss of fit indicated that the metric invariance held equal across the two waves. Subsequently, factor loadings and intercepts were set to be equal across time (χ^2^(649) = 2464.14, *p <* 0.001, CFI = 0.96, TLI = 0.95, AIC = 118, 615.25, BIC = 119,293.81, RMSEA = 0.05, SRMR = 0.03), which again did *not* worsen the model fit significantly (ΔCFI < 0.01); hence, scalar invariance was supported. Last, factor loadings, intercepts, and error terms were set to be equal across time (χ^2^(669) = 2499.82, *p <* 0.001, CFI = 0.96, TLI = 0.96, AIC = 118, 610.93, BIC = 119,185.09, RMSEA = 0.05, SRMR = 0.03), which again did *not* result in a significant loss of fit (ΔCFI < 0.01); hence, strict invariance was established. Considering that these subsequent tests did not lead to a significant decrease of fit, we can conclude that conventional levels of measurement invariance were achieved [70]. These results indicated that the study variables preserved their structure and meaning across the two waves, providing strong evidence for the methodological rigor of the study variables [70].

### 3.2. Structural Model Testing

The chi-statistics and the fit indices obtained for the alternative cross-lagged models are presented in Table 2, Table 3 and Table 4. First, we compared alternative models examining cross-lagged relationships between the study predictor “workplace changes” and the two study outcomes “learning outcomes” and “emotional exhaustion”. Both the causal (Δχ^2^ = 14.75, Δ*df* = 2, *p* < 0.001) and the reversed (Δχ^2^ = 14.75, Δ*df* = 2, *p* < 0.001) models fitted the data better than the stability model. Moreover, the reciprocal model had a significantly better fit than the causal and the reversed models (Δχ^2^ = 14.22, Δ*df* = 2, *p* < 0.001), indicating that the reciprocal model best represented the relationships between our predictor and the two study outcomes. 

Second, we tested cross-lagged relationships between the two mediators (i.e., learning demands and qualitative job insecurity) and the study outcomes (i.e., learning outcomes and emotional exhaustion). The stability model showed a worse fit compared to the causal model (Δχ^2^ = 69.74, Δ*df* = 4, *p* < 0.001) and the reversed model (Δχ^2^ = 34.20, Δ*df* = 4, *p* < 0.001), which fitted the data significantly better. In addition, the reciprocal model showed further improvement in model fit compared to the causal model (Δχ^2^ = 23.70, Δ*df* = 4, *p* < 0.001) and the reciprocal model (Δχ^2^ = 59.23, Δ*df* = 4, *p* < 0.001), again indicating that the reciprocal model fit the data best. 

Third, because no temporal effects were expected between the study predictor and the mediators, we tested normal, reversed, and reciprocal causation between the study predictor “workplace changes” at T2 and the study mediators “learning demands” and “qualitative job insecurity” at T2. Both the causal model (RMSEA = 0.03; CFI = 0.99; TLI = 0.99; SRMR = 0.04) and the reversed model (RMSEA = 0.02; CFI = 0.99; TLI = 0.99; SRMR = 0.03) showed a very good fit for the data. Here again, the reciprocal model fitted the data better compared to the causal model (Δχ^2^ = 32.78, Δ*df* = 1, *p* < 0.001) and the reversed model (Δχ^2^ = 10.36, Δ*df* = 1, *p* < 0.001). 

### 3.3. Hypotheses Testing 

Since the results suggested that the reciprocal model might be most suitable for explaining the relationships between the study variables, we tested a *reciprocal full-panel model* including direct and indirect effects. The fit statistics (χ^2^ (*df* = 622) = 2234.56; *p* < 0.001; RMSEA = 0.04; CFI = 0.96; TLI = 0.96) suggested a good fit for the data. 

Age and gender were included as control variables because prior studies have indicated that they can be meaningful co-variates of employee learning and well-being. Specifically, the literature suggests that female and older employees have better general well-being [77,78] and that training performance and perceived learning-relevant abilities may decline with age, while anxieties in learning situations may increase [79,80]. Altogether, studies have suggested that older workers may be less inclined to engage in learning and development activities compared to younger employees [81,82,83].

We analyzed the model twice, once *with* and once *without* the control variables (i.e., gender and age). In the model *with* control variables, gender and age were regressed on all study variables. The results showed only one significant relationship between the control and the study variables: age was negatively related to learning outcomes (*β* = −0.09, *p < 0*.001), indicating that older employees reported less learning at work. The outcomes obtained from the model with and from the model without control variables showed that there were no considerable differences in the overall findings of these two models. In line with Spector and Brannick’s [84] recommendation, for parsimony reasons, we chose to report the results for the model without control variables.

**Figure 1:**
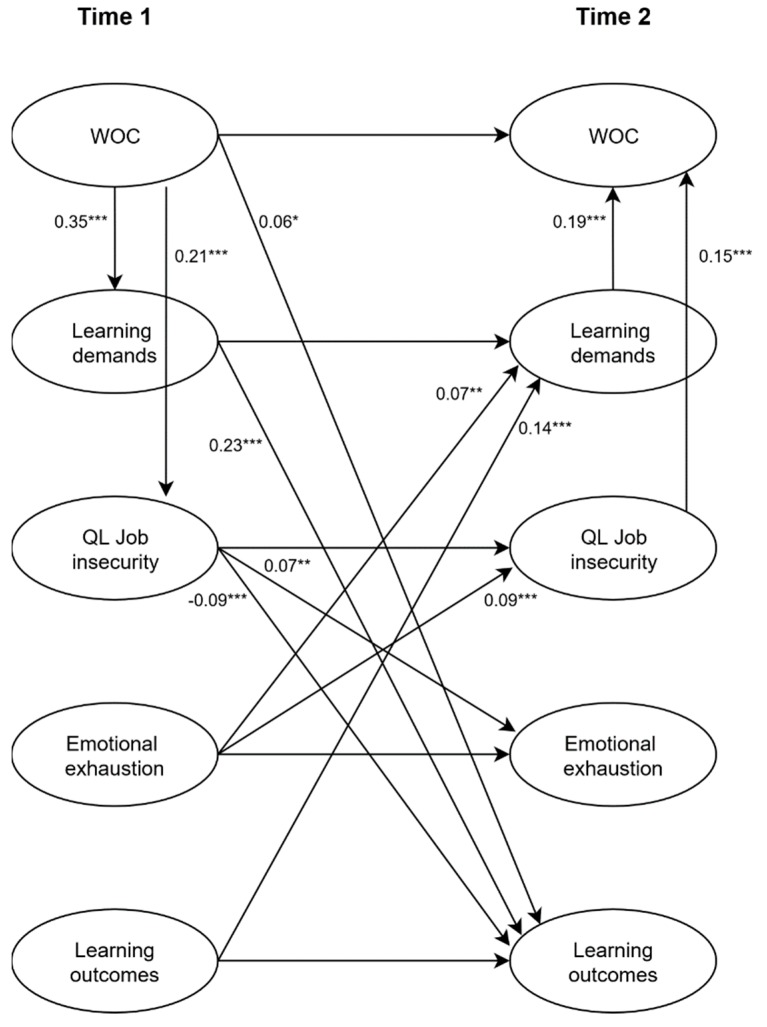
Reciprocal model with significant direct effects (*n* = 1366).

Direct effects. The regression coefficients of the structural paths obtained from the reciprocal model are presented in Figure 1. The results showed that workplace changes at T1 were positively and significantly associated with learning demands at T1 (*β* = 0.35, *p <* 0.001) and qualitative job insecurity at T1 (*β* = 0.21, *p <* 0.001), thus supporting Hypotheses 1 and 3. As expected, learning demands at T1 had a significant positive effect on learning outcomes at T2 (*β* = 0.23, *p <* 0.001), confirming Hypothesis 2a. However, the effect of learning demands at T1 on emotional exhaustion at T2 was not significant (*β* = 0.01, *ns*); thus, Hypothesis 2b was not supported. Qualitative job insecurity at T1 had a negative effect on learning outcomes at T2 (*β* = −0.09, *p* = 0.001) and a positive effect on emotional exhaustion at T2 (*β* = 0.07, *p* < 0.01), supporting Hypotheses 4a and 4b. In addition, workplace changes at T1 were positively and significantly associated with learning outcomes at T2 (*β* = 0.06, *p <* 0.05) but not significantly related to emotional exhaustion at T2 (*β* = 0.01, *ns*).

In addition to the hypothesized direct effects, several of the reversed causation relationships specified in the model were found to be significant. Specifically, learning demands at T2 (*β* = 0.19, *p <* 0.001) and qualitative job insecurity at T2 (*β* = 0.15, *p <* 0.001) were positively and significantly related to workplace changes at T2. Moreover, we found that learning outcomes at T1 were positively and significantly associated with learning demands at T2 (*β* = 0.14, *p <* 0.001) and that emotional exhaustion at T1 was positively and significantly related to learning demands at T2 (*β* = 0.07, *p* < 0.01). However, learning outcomes at T1 did not predict qualitative job insecurity at T2 (*β* = −0.002, *ns*), but emotional exhaustion did (*β* = 0.09, *p* = 0.001).

**Figure 2:**
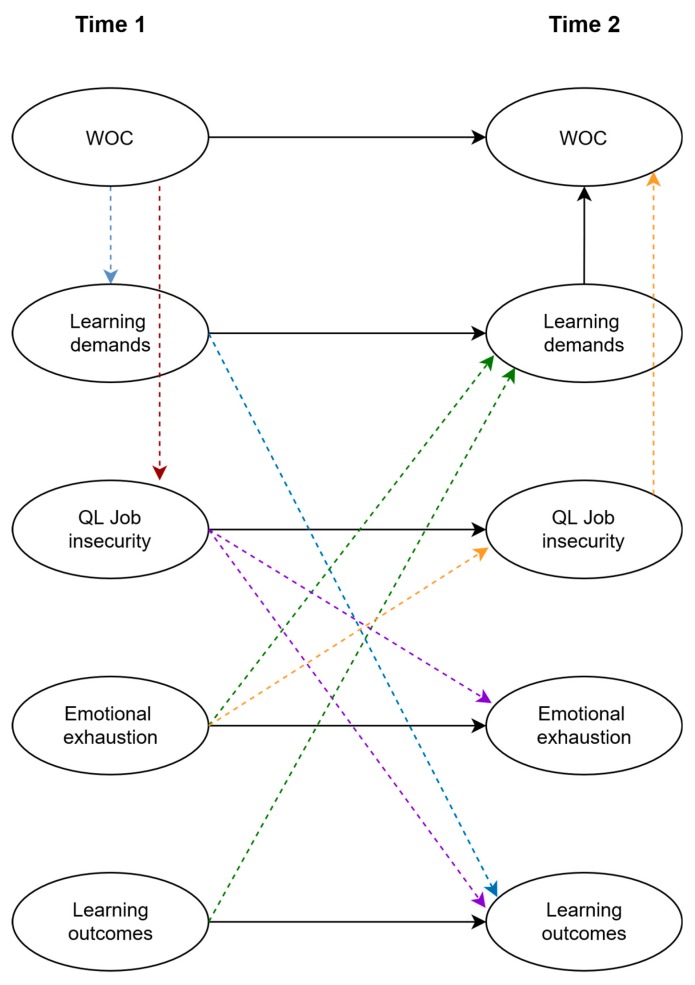
Reciprocal model with significant indirect effects (*n* = 1366).

Indirect effects. The results (see Figure 2) showed significant indirect effects of workplace changes at T1 through the mediator qualitative job insecurity at T1 on both learning outcomes at T2 (*β* = −0.02, *p* < 0.01) and emotional exhaustion at T2 (*β* = 0.02, *p* < 0.01). The indirect effect of workplace changes at T1 through the mediator learning demands at T1 was significant for learning outcomes at T2 (*β* = −0.03, *p* < 0.01) but not for emotional exhaustion at T2 (*β* = 0.01, *ns*). In addition to the indirect effects, workplace changes at T1 showed a significant direct effect on learning outcomes at T2 (*β* = 0.06, *p* < 0.05). There was no direct effect between workplace changes at T1 and emotional exhaustion at T2 (*β* = 0.01, *ns*), indicating that this relationship was fully mediated by qualitative job insecurity. 

In addition, several reversed causation indirect effects were found. Emotional exhaustion significantly related to workplace changes through qualitative job insecurity (*β* = 0.01, *p* < 0.01) and learning demands (*β* = 0.01, *p* < 0.01); learning outcomes related to workplace changes through learning demands (*β* = 0.02, *p* < 0.001) but *not* through qualitative job insecurity (*β* = −0.01, *ns*). Furthermore, no significant direct effect was found of workplace changes on emotional exhaustion (*β* = 0.03, *ns*), nor did we find an effect of workplace changes on learning outcomes (*β* = 0.02, *ns*). This indicated that qualitative job insecurity fully mediated the relationship between emotional exhaustion and workplace changes and that learning demands fully mediated the relationship between each of the two study outcomes (i.e., learning outcomes and emotional exhaustion) and the study predictor (i.e., workplace changes). 

In summary, the results lent considerable support to the suggested mediation processes: qualitative job insecurity fully mediated the relationship between workplace changes and emotional exhaustion, whereas learning demands and qualitative job insecurity partially mediated the relationship between workplace changes and learning outcomes. In addition to the expected normal causation relationships, several reversed indirect effects were established. The results showed full indirect effects from the study outcomes emotional exhaustion and learning outcomes by workplace changes through the mediator learning demands; also, qualitative job insecurity fully mediated the relationship between emotional exhaustion and workplace changes. 

## 4. Discussion

In this study, we integrated insights from the fields of organizational change and employee development. Our purpose was to investigate the positive and negative impact of workplace changes on employees. We argued that workplace changes do not uniformly influence the work context and employees, as they can cause a raise in challenging demands (learning demands) as well as in hindering demands (qualitative job insecurity), which in turn can affect employee learning and exhaustion over a six-month period. In line with our theoretical model, the findings showed that workplace changes affected employee learning outcomes by enhancing both learning demands and qualitative job insecurity, thus confirming Hypotheses H7a and H8a. At the same time, workplace changes activated perceptions of qualitative job insecurity, which resulted in increased levels of emotional exhaustion over time, thus confirming Hypothesis H8b. In contrast to our expectations, learning demands did not affect emotional exhaustion after a six-month period, thus Hypothesis 2b was rejected. In addition to the indirect relationships, we found that workplace changes were also directly related to learning outcomes but not to emotional exhaustion. Qualitative job insecurity thus fully mediated the relationship between workplace changes and emotional exhaustion, whereas learning demands and qualitative job insecurity only partially mediated the relationship between workplace changes and learning outcomes. 

The primary theoretical contribution of this study is its examination of workplace changes as a complex work stressor that can increase employees’ perceptions of the work context as being demanding (in both challenging and hindering ways), thereby shaping work environments that are conducive to learning and strain. The positive associations of workplace changes with qualitative job insecurity and learning demands (Hypotheses 1 and 3) indicate that workplace changes can boost work aspects such as challenge and hindrance stressors. Much research on organizational change in general, and workplace changes in particular, views change as a hindering work demand that can undermine employee well-being and increase absenteeism and voluntary turnover [12,46,47]. In line with Hypotheses H8b, the findings of this study support this claim, showing that workplace changes can trigger employee strain (i.e., emotional exhaustion) due to increased qualitative job insecurity. In other words, changes in the immediate work environment appear to instigate an energy-depletion process by enhancing employee perceptions of a potential threat to the continued existence of valued job aspects. Our finding that qualitative job insecurity fully carried the adverse impact of workplace changes on employee exhaustion suggests that sustaining the quality (i.e., the valuable aspects) of an employee’s job in times of workplace changes is of utmost importance for the employee’s well-being. It seems conceivable that anxious employees will report greater strain experiences, because when sustained over a prolonged period of time, work-related worries (e.g., of losing quality job aspects) can curtail a person’s energy (as change-driven negative emotions such as anxiety and frustration are deemed to trigger negative employee outcomes [2]). Our findings add to the job insecurity literature, as research underpinning the longitudinal consequences of qualitative job insecurity on work-related well-being is scarce to non-existing [28]. 

At the same time, in line with Hypothesis H7a, the findings showed that workplace changes can trigger a learning process. Changes in daily work tasks and routines imply increased learning demands as they constitute novel situations that require employees to modify and extend their prior knowledge and skills [17]. Moreover, employees who are experiencing skill shortages owing to workplace changes might be more motivated to learn because they need to update their competence [85]. Previous research already showed that challenge stressors are related to learning [5,48]. Moreover, researchers of workplace learning and management development have claimed that workplace changes can contribute to professional development and the acquisition of expertise necessary for employees’ adaptation to dynamic work contexts [18,19]. 

Our findings, however, also indicate that workplace changes can undermine employee learning owing to the negative impact of qualitative job insecurity (Hypothesis H8a). It is possible that employees who thought they were about to lose valued aspects of their job were less motivated to engage in learning activities. This was also the case in LePine et al. [5] study, where hindrance stress impacted learning performance through reduced learning motivation. As workplace changes and the associated insecurities would breach employees’ expectations for stability in the conditions of their employment [66,86], learning efforts made by the employee (that could benefit the organization) might be withheld as a means of keeping the relationship with the organization in balance [87]. Additionally, worries, stress arousal, and a lack of clarity concerning future job tasks may undermine effective learning, because of negative emotions and lack of clear learning goals [2,5]. This finding equally adds to the job insecurity literature, as qualitative job insecurity has not to date been studied in the context of learning, and its link with learning outcomes has not yet been analyzed. Altogether, it appears that workplace changes can serve as a double-edged sword for learning. While these changes can instigate a learning process by increasing learning demands, they can also activate certain job aspects (i.e., qualitative job insecurity) that can undermine employee learning. As such, our findings contribute to the limited empirical evidence on potential inhibitors of employee learning [5]. 

Our study highlights the potential of workplace changes to affect employee emotional exhaustion indirectly by increasing their perception of qualitative job insecurity. Our mediator learning demands, however, remained unrelated to emotional exhaustion after a six-month period. Various reasons might account for this finding. It is possible that the hindrance stressor qualitative job insecurity had a more dominant role in the occurrence of strain, overruling the potential influence of learning demands, a work stressor with potentially less health impact. Alternatively, learning demands might cause emotional exhaustion over a different time span than the six-month period covered in this study, as they could have a more immediate or short-term impact on emotional exhaustion. However, knowledge on the optimal time lag for assessing outcomes of learning demands is currently lacking. Longitudinal studies on learning and adaptation processes are still scarce, as studies using the challenge—hindrance stressors framework are predominantly cross-sectional [36,41] and focus on challenge demands other than learning demands (explored in this study). More longitudinal research is needed to investigate how change-related learning demands impact employees’ learning processes and exhaustion over time and to determine the optimal time lag for these associations. 

Moreover, in this study, we conducted a cross-lagged testing of the relationships in our model, which delivered several additional findings. As the reciprocal model showed the best fit for the data, some reversed effects were also observed. Learning outcomes were positively associated with the perception of learning demands over time, corroborating the idea that learning demands can be viewed as challenging. Exhaustion was positively associated with both learning demands and qualitative job insecurity over time. This finding is in line with previous research which also found reversed effects of health and well-being on job demands [88,89]. These results can be explained in at least two ways. First of all, exhaustion reduces the opportunities for recovery. This may impact performance at work, which could create additional demands later on [90]. As a consequence, exhausted employees might gradually move to a job with lesser quality over time, a phenomenon sometimes called the ‘drift hypothesis’ [91]. We could perhaps even assume that they may be exposed more often to workplace changes. Alternatively, exhausted workers might also perceive their work environment more negatively over time, which is sometimes labelled as ‘the gloomy perception mechanism’ [91]. This more negative evaluation could be expressed in aspects such as an increase in the perception of job insecurity and learning demands or the experience of workplace changes. Finally, it is interesting to note that learning outcomes were not related to qualitative job insecurity over time in this study. Acquiring new skills and competencies did not result in the reduction of job insecurity, which contrasts with previous views of increasing employability and skills as means of reducing job security [27]. 

Because of the established reversed causation effects, our study adds to the body of research that explores the influence of employee work-related well-being on (their perceptions of) the work context. We contribute to practice as our results suggest that organizations and practitioners should be mindful of the danger that energy-depletion processes might pose on the work environment of employees and their perceptions thereof as manageable, stable, and safe. This evidence may be viewed as extending theories as well (e.g., the Conservation of Resources (COR) theory [92] and the broaden-and-build theory [92]). COR assumes that loss spirals are triggered when individuals are confronted with suboptimal conditions that increasingly drain their resources. Our findings suggest that suboptimal psychological states may impact the (perception of) the environment as demanding and turbulent, thereby further draining a person’s resources. Moreover, it is possible that psychological work strain experiences (i.e., emotional exhaustion) may increase the perception of the environment as volatile (i.e., perception of rise in workplace changes) because energy depletion can narrow an individual’s cognitive functioning [93,94].

## 5. Limitations and Suggestions for Future Research

Like all research, this study is not without limitations. A first concern pertains to the use of self-reports, which can induce common-method bias. To address this issue, we thoroughly investigated the psychometric properties of our scales through a series of CFA tests; we provided evidence for the reliability of the scales, and the stability of the measurement model was tested over time, allowing us to control for baseline T1 effects of the variables. The results from these tests showed that the study scales have good psychometric properties and represented distinct measures of the separate constructs, suggesting that the findings are unlikely to be affected by common-method bias. Moreover, using employees’ perceptions of work stressors and their levels of learning and exhaustion is not uncommon in learning and stress research [43,44]. The perception of work stressors, learning, and exhaustion reflect subjective experiences and thus are best measured by surveying individuals. Still, future research may use other measures of employees’ well-being (e.g., sickness absence) and learning (e.g., manager reports on employee learning behaviours, obtained certificates, and total duration of trainings).

Furthermore, the choice of a six-month time lag for measuring the effects of learning demands and qualitative job insecurity on employee outcomes, as well as the decision to assess workplace changes and the related hindrance and challenge stressors synchronously, could raise questions. Stress theories (e.g., Lazarus & Folkman, [55]) suggest that evaluative mechanisms and responses occur almost instantly when a person is confronted with a stressor, which emphasizes the importance of looking at synchronous effects and following the respondents closely after the initial effects have occurred. Exploring different short time lags (such as a daily diary study or a week or two-week period) for measuring the effects of workplace changes on work stressors and outcome variables might increase our insight into the processes involved in workplace changes. In practice, determining the occurrence of a stressor such as workplace changes is often cumbersome, because the news about workplace changes usually circulate through informal networks long before the change is officially communicated. The results of this study may inspire further discussions and empirical research on the temporal effects of workplace changes on outcomes such as learning and well-being. Future research might use multiple (shorter and longer) time lags for testing the relationships between the variables of the current study in order to increase our understanding of the occurrence and duration of these effects. A better understanding regarding the timing and occurrence of stress effects during workplace changes is valuable for the planning of interventions that tackle these effects. 

Future research may also investigate the aspects of work context and change process that aggravate or counteract the insecurity experienced by employees during workplace changes. Change communication and leader–member exchange relationships have already shown relevance for employees’ responses to change [95]. Future studies may also focus on the role of individual differences, such as personality, adaptability, or emotion regulation [80], for employees coping with workplace change. These insights might be helpful in differentiating between target groups when developing person-tailored interventions. Researchers may also expand the current model by including more distal employee and organizational outcomes such as commitment, health, absenteeism, and turnover. The main drive for conducting this study (and for choosing this specific model) was to gain more insights into the processes that unfold from workplace changes. We thus focused on variables that could mediate these processes. Future research could explore the conditions under which workplace changes become more detrimental by, for example, analyzing whether learning demands and qualitative job insecurity moderate the relationship between workplace changes and employee outcomes (e.g., learning and strain). Additionally, studies could examine whether emotional exhaustion moderates the relationship between workplace changes and the study mediators learning demands and qualitative job insecurity. This could add to the limited knowledge on employee well-being as a pre-condition for dealing with the demands that workplace changes might generate; it is, for instance, possible that employees who feel overextended at work are more likely to experience workplace changes as harder to cope with and more taxing (i.e., higher levels of qualitative job insecurity and of learning demands) than happy and healthy workers. 

As the sample consisted of individuals who were employed in different companies and industries (a limitation inherent to this kind of panel-based data collection), we were not able to control for organizational interventions that might have occurred prior to or during the time of data collection. Such interventions might have, to some extent, affected our respondents and their experiences of both workplace changes and study outcomes. Despite this, there are advantages to testing our hypotheses on such diverse data. Testing our hypotheses on heterogeneous data demonstrated that the study concepts and, in particular, workplace changes as a job stressor (as operationalized in the current contribution) can occur in various organizational contexts and can impact employees (despite possible interferences of other simultaneously occurring organizational interventions). Moreover, our diverse data allow a broader generalization of the study findings.

Last, we conducted attrition analyses to test whether drop-out was completely at random or whether it could be linked to one or more of the variables included in this study. Our results showed that attrition among the participants in our sample was not at random with regard to job insecurity and emotional exhaustion. However, such a drop-out pattern is not uncommon for stress and strain research. Drop-out among individuals who are more strained and insecure is a well-known phenomenon in longitudinal work stress research [6]. 

## 6. Practical Implications

Our findings have also practical implications. Gaining a better understanding regarding the cost–benefit balance of workplace changes can be particularly valuable for innovation-oriented organizations that frequently implement workplace changes and place high value on employee learning [48]. Our results indicate that change-induced qualitative job insecurity can reduce employees’ energy and inhibit the learning processes needed to adapt to changes. Organizations could develop human resources management practices that are aimed at reducing employees’ worries about the future quality of their jobs when facing workplace changes. Organizations might also address the perceptions of qualitative job insecurity by providing a clear and timely communication about changes and by allowing some degree of involvement in decision-making when changes are being planned [56,78]. Management practices that enable employees to voice their concerns and be involved in the decision-making processes in times of change are deemed to boost positive outcomes, such as self-efficacy and well-being [56,96,97], and reduce negative outcomes, such as uncertainty and job insecurity [1]. Moreover, on the basis of our findings that change-related learning demands can increase employee learning, organizations are advised to support employees by providing ample time and opportunities for competence development. 

In conclusion, even though prior research has often viewed change as a source of negative outcomes, this study demonstrates that workplace changes should not be reduced to their negative consequences. While these changes can undermine employee well-being, they can also enhance employee learning over time. 

## Figures and Tables

**Table 1:** Means, standard deviations, and correlations among the study variables at the time points T1 and T2.

Scale	M	SD	1	2	3	4	5	6	7	8	9	10
1. WOC1	2.33	0.98	(0.80)									
2. WOC2	2.01	0.94	0.46	(0.81)								
3. LD1	2.85	0.99	0.32	0.28	(0.91)							
4. LD2	2.79	1.00	0.27	0.32	0.67	(0.92)						
5. QLJ1	2.64	0.93	0.18	0.15	0.09	0.07	(0.88)					
6. QLJ2	2.61	0.94	0.15	0.22	0.11	0.09	0.63	(0.89)				
7. LOS1	2.86	0.91	0.19	0.14	0.46	0.39	−0.18	-0.14	(0.95)			
8. LOS2	2.77	0.93	0.20	0.22	0.41	0.46	−0.12	-0.13	0.57	(0.95)		
9. EXH1	2.57	1.12	0.16	0.14	0.14	0.14	0.36	0.29	−0.10	−0.07	(0.93)	
10.EXH2	2.57	1.15	0.12	0.17	0.11	0.13	0.30	0.36	−0.09	−0.14	0.71	(0.93)
11. Gender	-	-	−0.11	−0.11	−0.19	−0.17	0.03	−0.03	−0.11	−0.09	−0.05	−0.03
12. Age	43.7	10.9	0.04	0.01	−0.11	−0.13	0.10	0.07	−0.24	−0.22	0.01	−0.01

Note: WOC = workplace changes, LD = learning demands, QLJIC = qualitative job insecurity, LOS = learning outcomes, EXH = emotional exhaustion; *r* = 0.05 to 0.07, *p* < 0.05., when *r* ≥ 0.08, *p* < 0.001.

**Table 2:** Fit statistics for investigating alternative models with combinations of the study predictors and outcomes (M1), and mediators and outcomes (M2).

Model	Model description	χ^2^	df	RMSEA	CFI	TLI	Model comparison	Δχ^2^	Δdf
Cross-lagged relationships between the study predictor workplace changes (WOC) and the two outcomes” learning outcomes” (LOS) &” exhaustion” (EXH)									
M1_mes_	Measurement	849.61	225	0.05	0.98	0.97			
M1_stabil_	Stability (autoregressive)	880.06	231	0.05	0.98	0.97			
M1_causal_	Causality (M1_stabil_ + WOC on LOS & EXH)	865.31	229	0.05	0.98	0.97	M1_stabil_ vs. M1_causal_	14.75**	2
M1_revers_	Reversed (M1_stabil_ + LOS & EXH on WOC)	865.31	229	0.05	0.98	0.97	M1_stabil_ vs. M1_revers_	14.75**	2
M1_recipr_	Reciprocal (M1_causal_ + M1_revers_)	851.09	227	0.05	0.98	0.97	M1_stabil_ vs. M1_recipr_	28.97**	4
							M1_causal_ vs. M1_recipr_	14.22**	2
							M1_revers_ vs. M1_recipr_	14.22**	2

Note: WOC = workplace changes, LD = learning demands, QLJIC = qualitative job insecurity, LOS = learning outcomes, EXH = emotional exhaustion; **p < 0.01; ***p < 0.001; *n* = 1366.

**Table 3:** Fit statistics for investigating alternative models with combinations of the study predictors and outcomes (M1), and mediators and outcomes (M2).

Model	Model description	χ^2^	df	RMSEA	CFI	TLI	Model comparison	Δχ^2^	Δdf
Cross-lagged relationships between the study mediators learning demands (LD) and qualitative job insecurity (QLJIC) and the two study outcomes learning outcomes (LOS) & exhaustion (EXH)									
M2_mes_	Measurement	1118.67	420	0.04	0.98	0.98			
M2_stabil_	Stability (autoregressive)	1220.50	432	0.04	0.98	0.98			
M2_causal_	Causality (M2_stabil_ + LD & QLJIC on LOS & EXH)	1150.76	428	0.04	0.98	0.98	M2_stabil_ vs. M2_causal_	69.74**	4
M2_revers_	Reversed (M2_stabil_ + LOS & EXH on LD & QLJIC)	1186.30	428	0.04	0.98	.098	M2_stabil_ vs. M2_revers_	34.20**	4
M2_recipr_	Reciprocal (M2_causal_ + M2_revers_)	1127.07	424	0.05	0.95	0.95	M2_stabil_ vs. M2_recipr_	93.43**	4
							M2_causal_ vs. M2_recipr_	23.70**	4
							M2_revers_ vs. M2_recipr_	59.23**	4

Note: **p < 0.01; ***p < 0.001; *n* = 1366.

**Table 4:** Fit statistics for investigating alternative models with combinations of the study predictors and mediators (M3).

Model	Model description	χ^2^	df	RMSEA	CFI	TLI	Model comparison	Δχ^2^	Δdf
Relationships between the study predictor WOC and the two mediators learning demands (LD) and qualitative job insecurity (QLJIC)									
M3_causal_	Causality (M3_stabil_ + WOC on LD & QLJIC)	280.50	151	0.03	0.99	0.99			
M3_revers_	Reversed (M3_stabil_ + LD & QLJIC on WOC)	258.08	151	0.02	0.99	0.99			
M3_recipr_	Reciprocal (M3_causal_ + M3_revers_)	247.72	150	0.02	0.99	0.99			
							M3_causal_ vs. M3_recipr_	32.78**	1
							M3_revers_ vs. M3_recipr_	10.36**	1

Note: **p < 0.01; ***p < 0.001; *n* = 1366.
